# State-of-the-Art in Skin Fluorescent Photography for Cosmetic and Skincare Research: From Molecular Spectra to AI Image Analysis

**DOI:** 10.3390/life14101271

**Published:** 2024-10-06

**Authors:** Konstantin Chekanov, Daniil Danko, Timur Tlyachev, Konstantin Kiselev, Ralf Hagens, Anastasia Georgievskaya

**Affiliations:** 1 Haut.AI OÜ, Telliskivi 60a/8, 10412 Tallinn, Harjumaa, Estonia; d.danko@haut.ai (D.D.); tlyachev@haut.ai (T.T.); a.georgievskaya@haut.ai (A.G.); 2 Beiersdorf AG, Beiersdorfstraße 1-9, 22529 Hamburg, Germany; ralf.hagens@beiersdorf.com

**Keywords:** skin fluorescence, skin fluorescence photography, follicular fluorescence, dermoscopy, computer vision, artificial intelligence, acne, hyperpigmentation, porphyrins, AGE

## Abstract

Autofluorescence is a remarkable property of human skin. It can be excited by UV and observed in the dark using special detection systems. The method of fluorescence photography (FP) is an effective non-invasive tool for skin assessment. It involves image capturing by a camera the emission of light quanta from fluorophore molecules in the skin. It serves as a useful tool for cosmetic and skincare research, especially for the detection of pathological skin states, like acne, psoriasis, etc. To the best of our knowledge, there is currently no comprehensive review that fully describes the application and physical principles of FP over the past five years. The current review covers various aspects of the skin FP method from its biophysical basis and the main fluorescent molecules of the skin to its potential applications and the principles of FP recording and analysis. We pay particular attention to recently reported works on the automatic analysis of FP based on artificial intelligence (AI). Thus, we argue that FP is a rapidly evolving technology with a wide range of potential applications. We propose potential directions of the development of this method, including new AI algorithms for the analysis and expanding the range of applications.

## 1. Introduction

Under illumination by UV or short-wavelength visible light, one can observe how human skin emits a bluish fluorescent glow [1,2,3]. Fluorescence is a remarkable optical property of the skin. This phenomenon can be observed and registered in the dark using special detection systems. In the literature, skin fluorescence is also denoted by the terms “skin autofluorescence” [2,3,4,5,6,7,8,9,10,11,12,13,14,15] and “skin intrinsic fluorescence” [16,17,18,19,20] to highlight the fact that it is caused by native structures and does not require specific fluorescent dyes.

Fluorescence of the skin was first documented in 1927 by Sigwald Bommer [21]. Specifically, he described the emission of blue light by the skin’s surface and dot-like orange emission corresponding to follicle openings under a Wood’s lamp. Even back then, the relationship between the character of the glow and physiological conditions of the skin were being discussed [21]. Throughout the years, knowledge about fluorescence of the skin has expanded. For its comprehensive study, a wide spectrum of chemical and physical instrumental methods was utilised, including in vitro studies of the fluorescent molecules of the skin by chromatography and/or mass spectrometry, fluorescence excitation and emission spectroscopy, hyperspectral imaging, two-photon microscopy, two-photon tomography, fluorescence lifetime imaging, and in vivo laser scanning confocal microscopy [5,9,13,22,23,24,25,26,27,28,29]. It had become increasingly clear that the skin’s autofluorescence signal was closely linked to biochemical processes there, whether during its normal development or in the case of pathologies. After almost a century, knowledge about skin fluorescence has evolved from phenomenological observations to a more detailed understanding of the processes at a molecular level. In 1967, Chalmers E. Cornelius III and George D. Ludwig first began to pay special attention to the orange–red skin fluorescence corresponding to comedones [30]. They determined the chemical nature of luminous molecules and found that they were produced by skin bacteria. The earliest studies of the molecular nature of the blue emission of the skin were conducted in the 1960s and 1970s. Zdeněk Deyl et al. have made significant contributions to the study of this phenomenon and age-related changes in the fluorescence of fibrillar skin proteins [31,32,33].

Fluorescence photography (FP) is a method based on capturing images of the skin under specific wavelengths of so-called “excitation light”—typically ultraviolet (UV) or blue—to observe patterns of fluorescence in situ. The FP technology of the skin was actively developed in the 1990s [9,23,24,34,35]. The first work on the FP of the skin was produced in 1996 by Luccina et al. [24]. Skin FP studies were pioneered by the Massachusetts General Hospital [35]. In particular, Nikiforos Kollias and co-authors used the existing basic knowledge about skin fluorescence to develop macro-imaging techniques. They proposed light filters for the selective detection of fluorescence from and excitation of different molecules [36]. UV-A-sensitive cameras were also introduced for the imaging [36]. Now, it is recognised as a prospective tool for diagnostics of various skin diseases from acne and hyperpigmentation to cancers. Furthermore, it is widely used for the evaluation of the effects of various cosmetics and medicines [22,23,34,35,37,38,39,40,41,42].

In the last two decades, a few reviews have focused on the different aspects of skin fluorescence [5,9,13,24,26,27,29,39,40,43]. Some of them have alluded to FP [1,9,24,26,29,40,44,45,46]. Recent scientific digests contain detailed but scattered information about skin FP. In particular, Pietkiewicz et al. [40] focus on detailed analysis of neoplastic and non-neoplastic dermatoses and their diagnostics using dermoscopic methods, including FP. Hollands et al. [44] discuss some applications of FP applying to oral conditions. Baig et al. [45] mention it among other methods of digital imaging and its applications in dermatology. In the review by Mojeski et al. [46], the methods of skin UV reflectance photography and UV-excited FP are compared. Their main applications in dermatology are also discussed. Wizenty et al. [29] examine different fluorescence-based techniques in dermatology. However, no new reviews—focused on FP and its theoretical foundations—have been published in the last five years.

In the current work, we aim to elucidate different aspects of the FP method, including its biophysical basis, the main fluorescent molecules of the skin, its potential applications in basic research, as well as in practical dermatology and cosmetology, as well as the principles of FP registration and analysis. Special attention is paid to recently published works on the automatic analysis of FP using artificial intelligence (AI).

For the review, we searched works using the Google Scholar (https://scholar.google.com/ (accessed: 1 April–30 May 2024) search engine with the following search queries: “skin fluorescence”, ([“skin fluorescence” ∨ ”fluorescence” ∨ “fluorescence photography”] ∧ [“NADP” ∨ “FAD” ∨ “FMN” ∨ “collagen” ∨ “elastin” ∨ “porphyrin”]), ([“skin fluorescence” ∨ “fluorescence photography”] ∧ [“sunscreen” ∨ “pigmentation” ∨ “hyperpigmentation” ∨ “melanoma” ∨ “ageing” ∨ “AGEs” ∨ “acne” ∨ “psoriasis”]), [“skin fluorescence” ∨ “fluorescence photography”] ∧ [“computer vision” ∨ “artificial intelligence” ∨ “machine learning”]). We preferred (but were not limited to) articles from the peer-reviewed journals indexed in Scopus and/or Web of Science. Special attention was paid to the recent publications (five years old and younger) as well as the high-impact articles cited in the field (50 and more citations, according to Google Scholar). Additional information was taken to elucidate general topics of interest (such as skin fibrillar proteins, skin chromophores, porphyrins, skin bacteria, and advanced glycation end-products) with the corresponding search queries.

## 2. Biophysical Basis of Skin Fluorescence

### 2.1. The Main Sources of Fluorescence in the Skin

**Figure 1:**
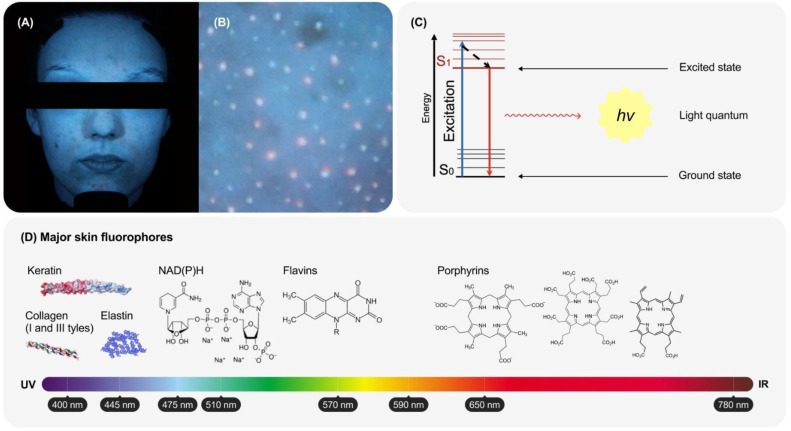
Fluorescent photographs (FPs) and physical principles of skin fluorescence phenomenon. Full-face FP captured with a VISIA-CR 5th gen (Canfield Scientific, Inc., Fairfield, CT, USA), Blue fluorescence mode-narrow band blue (405 ± 10 nm) strobes (**A**). Dermoscopic FP of the fragment of the skin (**B**). Jablonski diagram showing the physical principles of fluorescence quanta emission (**C**): the system (molecule or ion) absorbs the light and passes into an excited state from the ground state (S_0_), then it goes to the lowest excited state (S_1_) and emits the fluorescence light quantum (*hν*), blue and red arrows correspond to excitation and relaxation to the ground state of the system, respectively, wave arrow corresponds to the emitted light quantum, dotted line corresponds to the vibration relaxation to the lowest excited state. Main skin fluorophores located to scale of the visible light spectrum in accordance with their emission characteristics; R = adenosine diphosphate ribityl/phosphate ribityl (**D**).

There are various patterns of fluorescence in the skin, which are characterised by different intensities and colours [1,2,3] (Figure 1A,B). Reddish, greenish and white spots, as well as dark regions are observed on the blue background (Figure 1B). In general, the analysis of the spatial distribution of skin fluorescence in separate spectral channels provides useful information about skin conditions [43]. The fluorescence phenomenon is based on the following physical processes. Some molecules can absorb a light quantum and transition to one of the excited states with higher energy. Fluorescence is the emission of light as the result of a transition of the molecule (or ion) from the lowest excited state (S_1_) to the ground state (S_0_) without a change in spin multiplicity (Figure 1C). Phosphorescence (emission of light quanta with a change in spin multiplicity) is also possible in some molecules, but its quantum yield is very low. Alternatively, excited states are spent on chemical reactions or thermal dissipation through either internal or intercombination conversion [1,23,29]. 

**Figure 2:**
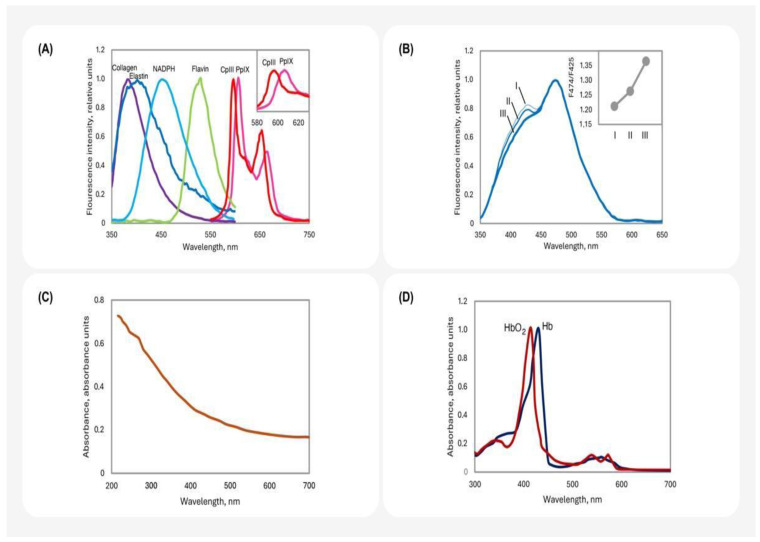
Spectral characteristics of compounds contributing to the skin fluorescence. Emission spectra of the main skin fluorophores in the visible range (adopted from [9] © De Gruyter Open Ltd. [50], © SPIE [51], © MDPI AG, with permission from [52] © Elsevier). Curves description: purple—collagen, dark blue—elastin, light blue—NADPH, green—flavins, red—coproporphyrin III, pink—protoporphyrin IX (**A**). Emission spectra of skin of different phototypes (I, II, and III); insertion: relationship F_474_/F_425_ as function of skin phototype (adopted with permission from [4] © Wiley) (**B**). Absorbance spectrum of eumelanin (adopted from [53] © SciEP) (**C**). Absorbance spectrum of free (Hb, red curve) and oxygenated (HbO_2_, deep blue curve) haemoglobin (adopted from [54] © SPIE) (**D**).

The colourful picture of skin fluorescence is ascribed to the palette of molecules emitting light (Figure 1D). The skin contains a number of such molecules (fluorophores) with different excitation and emission characteristics (Figure 2A). These characteristics are also affected by some parameters of the microenvironment, such as pH, viscosity, and the presence of quenchers [4,47]. Thus, the fluorescence spectrum of the skin is a superposition of the spectra of its fluorophores [4,22] (Figure 2B). Predominant emitters of the skin are aromatic amino acids, nicotinamide adenine dinucleotides (NAD(P)^+^ and NAD(P)H), collagen, keratin, flavins, and porphyrins [5,22,23,29,48,49] (Figure 1D and Figure 2A). Aromatic canonical proteinogenic amino acid residues, such as tryptophan, emit radiation in the UV range, so they will not be considered here. The total emission spectrum of the skin is characterised by strong radiation in the blue and blue–green regions. Collagen, elastin, and possibly NAD(P) contribute mostly to it [4]. It is important to note that most of the data on the spectral features of skin fluorophores have been obtained in vitro, and they can be different in vivo.

Spectral characteristics of skin nucleotides have been reported in many works. They are not different for NAD and NADP. Their fluorescence is excited and emitted in the bands of 340 ± 30 nm and 460 ± 50 nm, respectively [55] (Figure 2A). The fluorescence quantum yields of the reduced forms in 0.05 M Na-phosphate solution (pH 7.0) vary from 0.59% to 2.10%, depending on temperature [56]. The red fluorescence (580–640 nm) of oxidised flavin nucleotides (FMN^+^ and FAD^+^) (Figure 2A) is excited by irradiation from the visible range (450–500 nm) [6,34,50,57]. Its intensity is usually significantly lower than that of fluorophores in the near-UV region [57]. At standard pressure and temperature in H_2_O, the fluorescence quantum yields of FAD^+^ are 3.3% and 1.9% under excitation by 450 and 355 nm, respectively [47]. The reduced form, FAD∙H_2_, does not emit light [58]. Fluorescence quantum yields of FAD and NADH increase with the increase in the concentrations of CH_3_OH, C_2_H_5_OH, and CH₃CHOHCH₂OH. This means that the fraction of their excited states’ energy, utilised through non-radiative processes, is decreased.

The fibrillar proteins collagen and elastin are the main structural components of the skin. Collagens (type I and III) are major parts of the dermal extracellular matrix: at least 75% of its dry mass and 90% of its proteins are represented by collagen fibres [59]. Elastin is a glycoprotein. Its molecules form fibres and layers in the intercellular space of the dermis [60]. Some amino acid residues of collagens undergo changes as a result of post-translational modifications. This results in cross-linking between polypeptide chains and the formation of collagen fibres [61,62]. These cross-links are fluorophores of collagen molecules [31,32,33,48,63,64,65]. They can be conditionally divided into pepsin-digestible (excitation maximum at 335 nm and emission maximum at 390 nm) and collagenase-digestible (excitation maximum at 370 nm and emission maximum at 460 nm) cross-links [48]. In general, the listed fibrillar proteins of the skin are characterised by an absorption band at 300–340 nm and an emission at 420–460 nm [6,22,48,50,57] (Figure 2A). Although detailed studies providing values of quantum yields for these proteins are lacking, it appears that the quantum yields are higher for elastin than for collagen [66]. However, collagenase digestible cross-links contribute most to the fluorescence signal in the blue range [22].

Porphyrins are cyclic molecules containing four pyrrole rings as their structural basis. Fe^2+^-containing protoporphyrin (haem) forms complexes with various important proteins, such as haemoglobin (see below), myoglobin, and cytochromes. Although spectral properties may slightly differ among a wide variety of porphyrin molecules, they share some common features. Porphyrin compounds exhibit a strong absorption band in the blue region of the spectrum (the Soret band) as well as less-pronounced peaks at longer wavelengths (the Q bands). The maximum porphyrin fluorescence emission falls into the orange/red region of the spectrum (around 620–650 nm) [6,7,50,57,67,68,69,70] (Figure 2A). In vivo skin porphyrins have maxima et c.a. 600, 620, and 640 nm [7]. Protoporphyrin IX (PpIX) and coproporphyrin III (CpIII) exhibit red emission maxima at 635 and 620 nm, respectively [7,71]. PpIX is characterised by fluorescence quantum yields of 15.5% and of 1.1% in CH_3_OH and in 0.06 M Na-phosphate buffer (pH 7.0), respectively [72]. In whole blood at 25 °C, it is 7.0% [68]. The fluorescence quantum yields of 5.4% and 5.1% in 1 M HCl at standard temperature were determined for coproporphyrin I and uroporphyrin III, respectively [68]. The formation of complexes with metals can lead to an increase in the porphyrin quantum yield [69].

### 2.2. The Effects of Light Absorbance and Refraction on Skin Fluorescence

The effects of light refraction and absorption by the tissues affect the intensity of fluorescence at a certain wavelength as well as the shape of the entire spectrum [4,9,73]. Skin contains compounds with light-absorbing functional groups (chromophores) [22,37] (Figure 2C,D). They are responsible for optical shielding (screening), i.e., attenuation of the exciting light, and shielding of the fluorescence. Optic shielding is more pronounced at shorter wavelengths. The presence of inhomogeneities in the skin causes light refraction, resulting in reflection and scattering on small particles. To compensate fluorescence spectra for the effects of light scattering, reflection, and fluorescence spectra are recorded simultaneously for the skin sample. To calculate the compensated value of fluorescence intensity, the following expression is used:

(1) $$F\prime \left(\lambda \right)=\frac{F\left(\lambda \right)}{{\left(R_{ex}\right)}^{k_{ex}}\times {\left(R_{em}\right)}^{k_{em}}}$$

where $F\prime \left(\lambda \right)$ and $F\left(\lambda \right)$ are compensated and measured fluorescence intensities at the wavelength *λ*, respectively, and $R_{ex}$ and $R_{em}$ are the reflectances at the excitation and the emission wavelengths, respectively [18]. The exponents $k_{ex}$ and $k_{em}$ in (1) are selected in an empirical manner.

The infiltration of human skin by blood leads to an increasing local concentration of haemoglobin, which is a transporter of oxygen and bicarbonate in the organism. It is a complex protein consisting of four polypeptide chains, two α- and two β-type (so-called globin) and four haem prosthetic groups (Fe (II)-containing porphyrin rings). Haem is responsible for the binding of oxygen molecules [74]. At the same time, it plays the role of a chromophore [54]. Due to the Soret band, in the blue or violet region of the spectrum, causing its red coloration (Figure 2D). Notably, free haemoglobin and its complex with oxygen (oxyhaemoglobin) differ in spectral terms. It results in the colour difference of venous and arterial blood, which are enriched by haemoglobin and oxyhaemoglobin, respectively. In the first case, the colour is dark red (burgundy), and in the second case, it is bright red (scarlet) [54] (Figure 2D). The appearance of large blood vessels near the skin surface can be expected to contribute to the shielding of fluorescence. Indeed, local erythema on facial FPs correlates with the appearance of dark (non-fluorescent) areas [37].

Skin exhibits two types of melanin [75,76]. Eumelanin (black or brown) is produced in the course of oxidation of tyrosine (and/or phenylalanine) to *o*-dihydroxyphenylalanine (DOPA) and dopaquinone. Pheomelanin (yellow–red) is initially synthesised just like eumelanins, but DOPA undergoes cysteinylation either directly or by the mediation of glutathione. The end product of this reaction, cysteinyl–DOPA, further polymerises into various derivatives of benzothiazines [75,77]. Melanins are characterised by the effective absorption of light in the UV and blue regions [78,79]. Screening by these pigments leads to the appearance of a shoulder at 400–450 nm (Figure 2C), the severity of which depends on skin tone [4]. Thus, the shape of fluorescence spectra depends on skin tone (Figure 2B). Pronounced changes of the F_474_/F_425_ ration as a function of it are observed in the emission spectra (Figure 2B). Furthermore, ignoring skin tone in fluorescence analysis by automated systems can lead to a bias [80].

Overall, the skin’s fluorescence signal depends on (1) the intensity of the excited light, (2) the content, distribution and quantum yields of fluorophores, (3) the content of screening and shielding agents, as well as (4) the light-scattering properties of the skin. This signal carries valuable information about the presence and distribution of fluorophores and shielding agents there. It can reflect the physiological state of the skin, and therefore, fluorescence analysis is considered an important tool for diagnostics in medical and cosmetic applications.

## 3. The Main Applications of Skin Fluorescence

### 3.1. Assessment of Optical Screening

**Figure 3:**
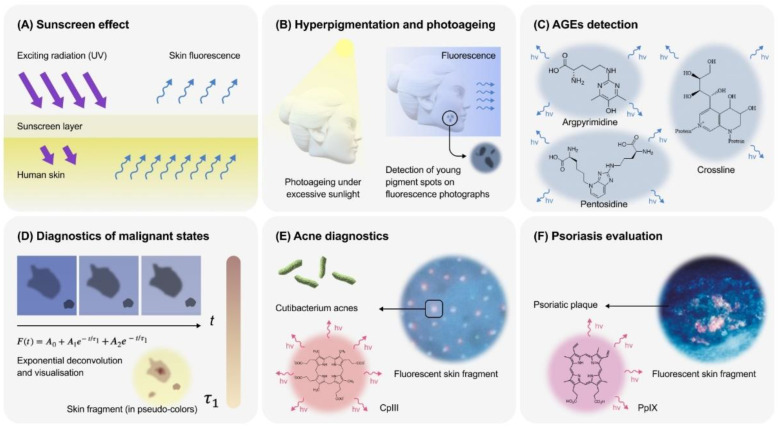
The main applications of the skin fluorescence phenomenon generated in Microsoft Office PowerPoint 2013 (Microsoft, Redmond, WA USA). Assessment of the sunscreen effect (**A**): exciting light and fluorescence are attenuated by the layer of sunscreen on the human skin, which reflects the effectiveness of light absorption. Assessment of hyperpigmentation of photoageing (**B**): pigment spots form under the sunlight, then become visible as dark spots in the fluorescence photographs. Detection of fluorescent advanced glycation end-products (AGEs) (**C**). Diagnostics of skin malignant states by the analysis of light-induced fluorescence decay (**D**): skin fragment is illuminated by the light for a certain period and fluorescence photographs are regularly taken, fluorescence intensity is registered in each pixel of the image as a function of time and deconvoluted by a bi-exponential function. Then, mapping of the image is performed based on the characteristic decay times, and pigmented malignant cells and healthy skin are characterised by the different characteristic times of the decay. Acne diagnostics (**E**): skin sebaceous follicles are colonised by *Cutibacterium acne*, which is responsible for the generation of coproporphyrin III (CpIII) emitting red fluorescence, which is seen as reddish spots in fluorescent photographs. Diagnostics of psoriasis (**F**): a pronounced pink–red emission is observed in the psoriatic plaque; the maximum at 635 nm corresponding to protoporphyrin IX (PpIX) is observed; the image of fluorescent psoriatic plaque is adopted from [88] © Elsevier.

The application of various screening agents appears to attenuate both the exciting light and emitted fluorescence. Various photoprotective agents, especially organic compounds of plant origin, such as the carotenoids β-carotene and astaxanthin [81] or mycosporine-like amino acids [82], are major components of sunscreens [83,84]. These compounds effectively absorb excess radiation (particularly UV, which is typically used for fluorescence excitation) to protect the skin against photodamage. Treatment of the skin with sunscreens leads to decreased excited fluorescence [46,85]. The effectiveness of skin sunscreens is commonly assessed by the sun protection factor (SPF). According to the Food and Drug Administration (FDA) [86], it shows how much UV solar radiation is required to produce sunburn on protected skin relative to the amount of solar energy required to produce sunburn on unprotected skin. Commonly, it is calculated as [87]

(2) $$SPF=\int_{280\;nm}^{400\;nm}\left(CIE\left(\lambda \right)\times E\left(\lambda \right)\right)d\lambda /\int_{280\;nm}^{400\;nm}\left(T\left(\lambda \right)\times CIE\left(\lambda \right)\times E\left(\lambda \right)\right)d\lambda$$

where *T(λ)* is the sunscreen transmission at the wavelength *λ*; *CIE(λ)* is the action spectrum value, recommended by the Commission Internationale de l’Éclairage (CIE) (erythemal spectral effectiveness), at the wavelength *λ*; and *E(λ)* is the spectral irradiance at the wavelength *λ*. It was demonstrated that measuring skin fluorescence excited by UV-A can be applied for the determination of SPF for the UV-A radiation [SPF(A)]. The expression of its calculation differs from (2) by the lower limit of integration set at 320 nm. Sunscreen application is accompanied by a decrease in skin autofluorescence proportional to SPF(A) calculated for irradiation by terrestrial midday midsummer sunlight for southern Europe (latitude 40°N, solar zenith angle 20°) as an E(λ) function. When the action of the cream is over, fluorescence intensity recovers to its initial level. Thus, it is possible to determine the period of sunscreen action and the time when it should be used again [85] (Figure 3A). The method is also applicable to assess the water resistance of a sunscreen [85].

Another application of evaluating integral skin autofluorescence in FP has been found in cosmetology. Facial fluorescent images were used to estimate the long-wear efficacy of foundations [89] (Figure 3A). In these experiments, the foundation (SPF 30) was gradually removed from the face. On the one hand, organic and inorganic compounds of the foundation filtered exciting UV radiation. On the other hand, the iron and titanium oxides in its formulation weaken the fluorescence signal. The percentage of remaining foundation, calculated based on the values of the fluorescence brightness extracted in the green channel, correlated with the coverage grading scores [89].

### 3.2. Hyperpigmentation and Photoageing

FP shows collagen fluorescence areas as blue light areas and areas of melanin pigmentation as dark spots due to fluorescence shielding (Figure 1B) [25]. Such images display areas of hyperpigmentation with much greater clarity than visible light photography. Indeed, tracking treatment-related changes in hyperpigmentation under bright-field conditions, especially in the early stages when changes are subtle, can be challenging [38]. In studies on the treatment of diffuse hyperpigmentation with tretinoin, FP analysis revealed a significant decrease after treatment, while most pigmented macules were observed on bright-field photographs neither before nor after treatment [38]. The analysis of skin FP was applied to evaluate the signs of pheomelanin-enriched facial hyperpigmentation: the spotty perifollicular type, the accretive globular type, and the elongated type of the sunny side of wrinkles [90]. Based on this study, a three-week application of azelaic acid and soy extract led to the decrease in pigsigns of shielding in fluorescence images and significant skin lightening [90]. The higher sensitivity of the FP method makes it a promising tool in the arsenal of current instrumental dermatology and cosmetology for hyperpigmentation analysis (Figure 3B).

Photoageing is a complex of destructive processes in the skin due to prolonged exposure to excess light. It results in the diverse undesirable effects: high skin dryness, lacking elasticity, hyperpigmentation, superficial and deep wrinkles [91]. Fortunately, it is possible to diagnose the symptoms of photoageing before its visible manifestation. The changes in the structure of the network of collagen fibres is one of the most noticeable biochemical markers of photoageing [85]. As was shown, collagen fluorescence correlates with age [92]. It was demonstrated on the murine model that ageing was accompanied by the change in the ratio of fluorescence peaks at 340 and 360 nm. They corresponded to pepsin- and collagenase digestible cross-links of collagen, respectively [38,93]. Under the chronic UV-B radiation, 340 and 360 nm bands became indistinguishable, and additional emission maxima appeared in the UV range [38]. Chronic UV-A radiation in mice was also accompanied by a gradual decrease in height of the emission peak of the pepsin-digestible links [93]. This indicates that photoageing is accompanied by reproducible changes in the fluorescence spectra of skin fibrillar proteins and other fluorophores. Most likely, pepsin-digestible collagen links are one of the main targets of UV action. Thus, the evaluation of collagen fluorescence may be useful to detect the changes caused by photodamage (Figure 3B).

The effect of shielding of blue fluorescence by pigments can be utilised to detect other skin lesions characterised by specific patterns of hyper- or hypopigmentation. For example, dark areas in fluorescent images, corresponding to hyperpigmentation, are observed in conditions such as melanocytic naevus and Becker’s naevus [40]. The opposite phenomenon, hypopigmentation, appears as enhanced fluorescence in areas of skin lesions surrounded by normal skin emitting light at a lower intensity. This is typical of certain dermatoses, such as progressive macular hypomelanosis or vitiligo [94,95]. Pityriasis versicolor, caused by *Malassezia* spp. (Basidiomycota), manifests as either hypo- or hyperpigmentation. The affected zones are characterised by light-green or dark-green fluorescence, respectively, due to the presence of fungal fluorophores [40].

### 3.3. Age, AGEs and Skin Fluorescence

Advanced glycation end-products (AGEs) are a diverse group of compounds of biological origin that spontaneously form as a result of non-enzymatic Maillard reactions between keto-/aldehyde and amino groups of nucleic acids, free amino acids, peptides and lipids [94,95]. They can form in the organism (endogenous AGEs) or originate from food (exogenous or dietary AGEs) [94]. Some AGEs, called “non-toxic AGEs”, form as a result of the normal functioning of the organism (post-translational modifications of proteins and neutralisation of excess aldehyde/carbonyl compounds). However, there are so-called “toxic AGEs”, which activate intracellular pro-inflammatory pathways, generate cytotoxic reactive oxygen species, and impair mitochondrial function. The latter deserve special attention since their high content is associated with a number of pathological states: non-alcoholic fatty liver disease, non-alcoholic steatohepatitis, cardiovascular diseases, infertility, Alzheimer’s disease, arteriosclerosis, and cancer. They can also be involved in the acceleration of the ageing process due to glycation-related protein damage [94,96]. In particular, the glycation of long-living fibrillar proteins of the extracellular matrix, such as collagen, leads to their agglomeration affecting the state of the skin [65,94,96]. The pro-inflammatory effect of AGEs also promotes ageing [94].

Some AGEs, such as pentosidine, crossline and argpyrimidine, can emit a detectable fluorescence [94,97,98,99] (Figure 3C), so they may be considered as one more skin fluorophore. There are little data regarding the spectral characteristics of AGEs. Their excitation maxima lie in the UV-A range [49,100,101]. The emission peaks of the modified forms of human serum albumin fall within the range of 350–450 nm with a shoulder continuing to 550 nm [101]. Notably, when excited by 320 nm radiation, the emission maximum of the modified forms is observed at 395 nm (UV-violet range), whereas when excited by 365 nm radiation, it is blue (440 nm) [101]. In addition, excitation by the blue light (450 nm) leads to an emission with the maximum in the 515–535 nm range. This is observed for the modified arginine but not for the modified lysine [101]. The peak of 350 nm excited fluorescence of collagen modified with ribose is observed in the range of 400–500 nm (using the 405 nm cut-off for light filters) [102]. The intensity of this fluorescence increases with the time of incubation with ribose [102]. It should be noted that while a fluorescence signal is suitable for detecting AGEs, it does not always correlate with their actual content [101]. The level of AGEs in healthy subjects aged 20–60 years correlated with 440 nm light with the emission maximum at 520 nm, but this was not the case with violet and blue fluorescence excited by UV [103]. The fluorescence quantum yield of the modified haemoglobin is c.a. 19% when excited by 308 nm UV light with an emission maximum at 345 nm [100]. Notably, AGEs’ fluorescence is observed even in the presence of haem as a quencher [100].

The analysis of skin fluorescence is the main method for the in vivo assay of AGEs level (Figure 3C). Non-invasive spectral methods of fluorescence assessment of skin [14,65,102,104] provide more precise information. They are based on the evaluation of the shape of the fluorescence spectra [49,65,104]. For instance, the AGE Reader (DiagnOptics Technologies BV, Groningen, The Netherlands) is widely used for the non-invasive spectral analysis of AGEs’ fluorescence in the skin [17]. It was applied to detect the increase in their level in patients with peripheral artery disease [16], diabetes [13], metabolic syndrome [15], atherosclerosis [20] and many other studies. Not only the spectral shape but also integral intensity (area under the curve in a certain spectral range) are used [18,19]. Due to the distinguishing spectral properties of different AGEs and the overlapping of their spectra with the spectra of other skin fluorophores, it is especially important to compensate fluorescence spectra for the effects of light scattering [18]. For this purpose, spectrometers for AGE detection are often equipped with the system for registration of the skin reflectance spectra. In this case, the compensated fluorescence intensity is calculated using Equation (1).

More specialised methods include fluorescence lifetime analysis after switching off the excitation light. Fukushima et al. [102] deconvoluted 370 nm excited fluorescence of collagen incubated with ribose into the multi-exponential kinetics in the nanosecond range:

(3) $$F\left(t\right)=\sum_{i=1}^{N}\left(A_{i}e^{-t/\tau_{i}}\right)$$

and determined the average fluorescence lifetime (τ_A_) calculated from (3) as

(4) $$\tau_{A}=\sum_{i=1}^{N}\left(A_{i}\tau_{i}\right)/\sum_{i=1}^{N}A_{i},$$

where $F\left(t\right)$ is fluorescence intensity as a function of time, *t* is time, $A_{i}$ are constants in units of fluorescence intensity and $\tau_{i}$ are characteristic fluorescence lifetimes. 

The values of *τ_A_*, calculated for *N =* 3 in (3) and (4) decreased with the time of incubation, while for *N =* 2, *τ_A_* remained constant at a c.a. of 1.6 ns [102]. Thus, the data on fluorescence lifetimes provide essential information about the level of glycation during the AGE formation.

The presence of fluorescence from substances other than AGEs obscures the outcomes [98]. Due to insufficient resolution, the use of the FP method for AGE assay is challenging and, therefore, it has not yet found wide application in this area. However, Larsson et al. [105] took an attempt to implement it using a system of light filters and a special procedure of image analysis.

### 3.4. Skin’s Malignant States

Although in the earliest works, an autofluorescence signal was not considered as an appropriate diagnostic tool for skin malignant states [106], now it is generally accepted that the evaluation of pigmentation patterns by the FP method could be helpful in this task [34]. Skin fluorescence spectroscopy and FP have found application in the diagnostics of skin cancers (Figure 3D). Skin cancer, including melanoma, can often present as changes in pigmentation or the appearance of other lesions on the skin. These changes can sometimes be a sign of skin cancers [107]. Moreover, due to the presence of yellow bilirubin fluorescence, it is possible to detect ulcerations typical of skin cancers [40]. Autofluorescent skin imaging was also proposed for the control of tumour margins in the non-melanoma skin cancers [8]. The analysis of fluorescent images helps to reveal subclinical pigmentation for the revealing of the discrete melanoderma: detection of patchwork of speckled darker dots and larger globular macules [34,108]. Basal cell carcinoma is characterised by aggregated yellow globules and clods with an intense white emission in fluorescent images. [40]. In 1991, the method of melanoma diagnostics based on skin FP was patented by the Phillips Corporation [109]. 

Considerable success was achieved in skin cancer diagnostics using the fluorescence photobleaching technique [9]. This phenomenon lies in the decrease in fluorescence intensity under constant irradiation by a laser [73]. This effect can serve for several days [9]. Fluorescence decline as a result of photobleaching is well explained by the bi-exponential function

(5) $$F\left(t\right)=A_{0}+A_{1}e^{-t/\tau_{1}}+A_{2}e^{-t/\tau_{2}}$$

where *F(t)* is the fluorescence intensity as a function of time (*t*), *A*_0_, *A*_1_, and *A*_2_ are the constants in units of fluorescence intensity, and τ_1_ and τ_2_ are the characteristic times of fluorescence decline. Thus, there are two components of the laser-induced decrease: fast with the times lower than a minute (*τ*_1_) and slow with the times several minutes and more (*τ*_2_) [9]. Although the exact mechanisms of photobleaching in human skin remain unclear [9], there is a correlation between the parameters of decline kinetics and hyperpigmentation. In each photograph, the parameter *τ*_1_ increases with the increase in local melanin content. Pigmented skin naevi contain higher melanin concentrations than surrounding tissues. Therefore, melanin can act as an agent that slows fluorescence bleaching [11]. The rate of fluorescence decline can also decrease due to the presence of haemoglobin chromophores, as in the case of angioma [12]. The nature of the *τ*_2_ component is less understandable, whereas, to a certain degree, it also reflects the patterns of high melanin content [11]. However, in terms of clinical applications its value has to be studied in further works. Collectively, the analysis of fluorescence photobleaching makes it possible to reveal pigmentation patterns hidden on the bright-field images [11]. This method can detect even areas with low melanin content and evaluate the non-uniformity of pigmentation, which is important in the diagnostics of skin’s malignant states [10]. The possibility of the use of skin fluorescence bleaching was tested for the analysis of angioma, dermatofibroma, basalioma, giant congenital naevi, papillary naevi, hyperpigmented naevi, dysplastic naevi, and blue naevi [12].

Collectively, the skin FP method helps to reveal malignant neoplastic dermatoses at an early stage, assess their post-treatment progress, and identify biopsy sites [8,9,11,12,40,106,107].

### 3.5. Follicular Fluorescence and Acne

A couple of types of exocrine glands are abundant in human skin. They include sweat and sebaceous glands. The latter are organised into complex structures, i.e., sebaceous or pilosebaceous follicles. These glands communicate with the external environment through pores. Sebaceous glands located in the follicles produce skin sebum for maintaining the health and integrity of the skin and hair, providing hydration, protection, and the regulation of various physiological processes [110].

Normally, a human organism contains, in small amounts, the products of haem metabolism, including its direct precursor, PpIX, and some of them can appear in the pilosebaceous follicles [111]. In some cases, protoporphyrins can be spontaneously oxidised to corresponded porphyrins. This leads to the irreversible removal of the latter from the metabolic pathway and their accumulation in the organism [67,111]. These follicles, in turn, are often colonised by the bacterium *Cutibacterium acnes* (Actinomycetes, Propionibacteriaceae), which was formerly known as *Propionibacterium acne* (Figure 3E). This microorganism is a part of the normal microbiota of the skin. However, in abundance due to metabolic shifts, they can induce specific inflammatory reactions, promoting the development of acne, which is a common skin disorder that occurs in hair follicles and manifests as comedones (blackheads and whiteheads), papules, pustules, and, in the more severe cases, as cysts and nodules [24,67,112]. As a result, *C. acnes* in the follicles forms common metabolic pathways with humans, leading to the generation of a large amount of porphyrin [30,67,113,114]. In the microaerobic conditions of skin sebum, they oxidise a fraction of the porphyrinogen’s pool to uroporphyrin III and CpIII instead of PpIX [1,30,67,115] (Figure 3E). CpIII is a predominant fluorophore of *C. acnes*, and its content correlates with the number of colony-forming units of the bacterium [116]. In FP, clinical signs of acne correlate with CpIII-associated but not PpIX-associated emissions [117]. Because of the presence of these molecules, pilosebaceous follicles can emit an orange–red light [1,7,22,24,118]. This phenomenon is called follicular fluorescence (FF) (Figure 3E).

Bacteria-produced porphyrins are effective photosynthesisers [7,34,39,72]: 407 nm irradiation of isolated *C. acnes* colonies led to a decrease in fluorescence level in the characteristic bands of their porphyrins (at 635 and 700 nm) and an increase in the emission near 670 nm, corresponding to putative photoproducts [7]. These products also differed from the native porphyrins in terms of the parameters of the bi-exponential deconvolution of fluorescence decay (namely, fluorescence lifetimes) [7]. The treatment of patients with Asian-type skin by the intense pulsed light (530–750 nm) with or without the use of topical methyl aminolevulinate (a porphyrin precursor) led to a decrease in non-inflammatory skin lesions [119]. This gives grounds for considering photodynamic therapy as a promising treatment for acne.

FF plays a very important role in acne diagnostics (Figure 3E). It reflects the qualitative and quantitative composition of sebum [118,120]. In 1994, Elizabeth Arden Co. and Division of Conopco Inc. patented the method of acne diagnostics based on FP captured using the system of light filters. It is likely that FF from open comedones is the only type observed in FPs [24]. Acne evaluation appears to be the primary application for skin FP technology. Here, just a few cases for its use are presented. Treatment of patients with acne with benzoyl peroxide (BPO) led to the visual decrease in reddish spots and the gradual decline of numerically estimated integral intensity of FF on dermoscopic photographs [41,117]. Discontinuation of the treatment led to the recovery of the FF intensity [41]. It should be noted that the CpIII-associated fluorescence is more affected by the BPO treatment than the PpIX-associated fluorescence [117]. Treatment by clindamycin is accompanied by a visual decrease in FF in the photographs [37]. Treatment with a combination of clindamycin and BPO led to a decrease in CpIII and PpIX fluorescence to approximately the same degree. It was also accompanied by a decrease in acne manifestations [117]. Treatment of acne with 1% *Trachyspermum ammi* (ajwain) fruits essential oil led to a decrease in the number and size of spots, corresponding to FF in the photographs. It was accompanied by reduction in the total and non-inflammatory lesions of the skin [42]. The level of CpIII fluorescence and the abundance of *C. acnes* decreased immediately after 1 h of swimming [121]. Reddish FF is not the only sign of acne. Its complications also include vasodilation, usually leading to local increases in extracellular fluid in the dermal tissue (oedema), manifested as a raised lesion (papule), depositions of excessive sebaceous exudate with dead neutrophils (pustules), as well as local hyperpigmentation [24]. The method of FP enhances the contrast of skin pigmentation, which is also important for acne detection [24].

Although FF is generally recognised as a reliable indicator of acne, a few criticisms of its use can be found in the literature. Therefore, it is important to discuss possible pro et contra the limitations of the applications of the FP method for diagnostics this skin lesion.

Firstly, whether the colonisation of *C. acnes* is the sole source of the orange–red FF remains a subject of debate. On one hand, some authors report that fluorescence data correlate with the counting of *C. acnes* colony-forming units obtained from patients after reseeding [41,122,123]. Moreover, it was shown that SZ95 sebocytes and isolated sebaceous glands did not emit light, while *C. acnes* bacteria isolated from the follicles and their porphyrin extracts showed emission with a maximum at 332 nm, corresponding to CpIII [71,124]. CpIII fluorescence correlates with the level of amplicons of *C. acnes* in the 16S rRNA metabarcoding data [121]. On the other hand, there are works suggesting the FF level reflects the sebum level rather than the amount of *C. acnes*. This raises questions about other sources of orange–red fluorescence in the skin. Youn et al. [125] did not find a significant correlation between the level of red fluorescence area on the photographs and the rate of *C. acnes* isolation. However, conflicting interpretations yielded from the results in [71,125] could be explained by the difference in the protocols of isolation and culturing of the bacteria. According to [120,126,127,128] and other works, the levels of FF correlate with the sebum levels, but in these cases, the authors did not study the *C. acnes* numbers. The bacterium *Staphylococcus epidermidis* (Bacilli, Staphylococcaceae), isolated from the follicles, also emits red fluorescence, but with the maximum at 320 nm, indicating CpIII [71]. It demonstrates differences in the ratios of porphyrin fluorophores across bacteria. *S. epidermidis* is the most abundant bacterium in human skin, and it is generally recognised as a commensal not involved in acne development [129,130]. Furthermore, it competes with *C. acnes* and prevents the over-colonisation of the follicles by the pathogens [130]. Therefore, this bacterium also can contribute to the fluorescence signal, and it should be taken into account in the analyses of FPs for acne diagnostics.

Secondly, the analysis of FF must take into account the individual characteristics of the patients and the specific body regions studied. Porphyrin fluorescence is irregularly distributed over the skin, reflecting the patterns of acne appearance. On the face, its highest level is observed in the so-called T-zone (chin, forehead and nose) for both males and females [120,122,125,126,128]. The basal porphyrin content in healthy subjects strongly varies across healthy people [70]. For instance, the total level of FF is the highest in adults aged 20–50 years and decreased after the age of 50 years [122,131]. The age distribution of fluorescence can be explained by the level of sebum secretion, which is the highest in the group aged 25–40 years [132]. As a nutrient-rich microaerobic compartment, sebum, in turn, may be colonised by higher numbers of *C. acnes* and may exhibit higher fluorescence signals. In addition, individuals also differ in terms of their basal PpIX contents [133].

Thirdly, the shape of the free coproporphyrin and uroporphyrin spectra (which in terms of image analysis corresponds to the colour) in vitro strongly depends on the microenvironment parameters, such as pH and the presence of other organic compounds [132,134]. There are little data on the white, blue, yellow and even green fluorescence of pilosebaceous follicles. The nature of this phenomena is not completely understood [24,26,40,118,123,128,135]. Nevertheless, CpIII spectra registered in vivo in the *C. acnes* culture were similar to that in vitro in 3M HCl. The emission of *C. acnes* cells cultured in the pH range of 3.1–6.1, with and without the addition of human sebum, did not change [71]. One can speculate that the appearance of fluorescence other than red can be explained by alien substances from cosmetics used by individuals. For instance, betalains, common components of some skincare products [136], emit radiation in the range of 530–600 nm [137]. The diversity of colours in the images can reflect the variety of breakdown products of porphyrins and other organic compounds. Herpens et al. speculated that green FF is due to accumulation of flavins [138]. Unfortunately, there is no solid evidence supporting these hypotheses. Moreover, the sebum colour could be subjectively perceived by the operator and may vary depending on the camera used [135]. Blue FF is a diagnostic sign of Malassezia folliculitis caused by *Malassezia* spp. [40]. Red fluorescence is absent in areas of its colonisation due to the antibacterial effect of azelaic acid, produced by these fungi. Meanwhile, enhanced blue FF is observed due to hyperkeratosis [40].

Collectively, in the application of FP for, e.g., the evaluation of a treatment against acne, it is better to be guided by the percentage of the emission level for each patient than by the average absolute values in the population.

### 3.6. Psoriasis

Psoriasis is a chronic non-infectious skin disease expressed as papules (red, excessively dry spots raised above the skin surface). They merge with each other, resulting in plaque formation. Papules are sites of chronic inflammation characterised by excessive angiogenesis and an excessive proliferation of lymphocytes, macrophages, and keratinocytes. Affected areas of the skin are characterised by excess desquamation [139].

A pronounced pink–red emission can be observed in these lesions on the FP. Spectral analysis reveals the maximum at 635 nm, corresponding to PpIX (as opposed to acne when CpIII is predominant) [40,88,140,141] (Figure 3F). The presence of this compound was supported by high-performance liquid chromatography [88]. However, it is not observed in all patients with the plaque [88,140]. This emission is related only to stratum corneum and is not detected in other skin layers [88]. In the photographs, red fluorescence is observed only in more severe lesions. Severity, as scored in terms of the Psoriasis Severity Index [142], correlates with the PpIX fluorescence intensity [140]. The origin of the high concentrations of PpIX in the stratum corneum from the zones of psoriatic plaque remains unclear [141]. It can be of either microbial or non-microbial origin. However, a pink–red signal on the fluorescence images may be used as a tool for the diagnostics of the psoriasis and assessment of its severity (Figure 3F).

It should be noted that some skin lesions can be accompanied by red, blue, and yellow non-follicular fluorescence; however, these occurrences are less discussed in the literature. In addition to psoriasis, red emission is observed in conditions such as erythrasma and pitted keratolysis [40]. Yellow-green fluorescence can be attributed to trichobacteriosis axillaris or infections caused by *Pseudomonas aeruginosa* (Gammaproteobacteria, Pseudomonadaceae), which produces the fluorophore pyoverdine [40].

## 4. Devices and Registration Principles

### 4.1. General Principles of FP Registration

It is important to distinguish between UV-reflectance photography and UV-FP. The UV-reflectance technique aims to register UV radiation reflected by the skin on a UV-sensitive camera [40,46,143,144]. This method is used to study patterns of pigmentation or erythema due to high extinction coefficients of skin chromophores in the UV range [46,144]. It is also applicable for studies of sunscreens [46]. However, it is impossible to observe, e.g., an acne-related red emission. In this case, it is necessary to install a long-wavelength filter in front of the camera lens to remove a fluorescent signal in the visible range [46]. At the same time, in UV-FP, this filter should cut off exciting UV to remove a signal from its reflection [46]. Hybrid systems, registering both reflection and fluorescence, are also useful [46].

**Figure 4:**
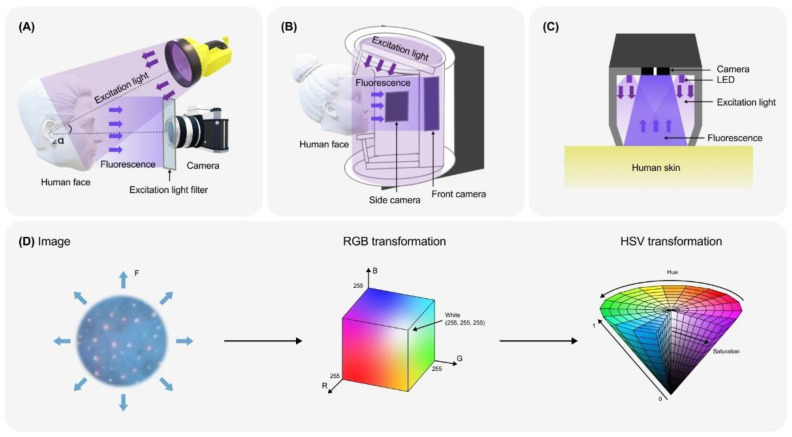
The principles of registration of fluorescent photography and data analysis generated in Microsoft Office PowerPoint 2013 (Microsoft, Redmond, WA, USA). The simplest scheme for the registration of skin fluorescence, including a source of excitation light and detection of fluorescence by a photocamera; a light filter cutting off the excitation light is located beyond the camera lens, α is the corner between the direction of excitation light and the main optical axis of the camera (**A**). The standardised system for full-face registration (**B**). The standardised system for registration of dermoscopic images (**C**). The procedure of data analysis: a digital image is detected, fluorescence intensity is associated with luminance, and the analysis in the RGB coordinates can be performed—then, it is possible to transfer the image to other colour spaces, such as HSV (**D**).

The simplest—and, historically, the first—experimental set-ups for the observation of skin fluorescence and FP taking (Figure 4A) include the following components. The first is the sample (human skin). To the best of our knowledge, there are no conventional strict protocols of individuals’ preparation for FP registration. Usually, they include makeup removal, requirement for a special cleansing or the band on its use (depending on the study), and ethical considerations. Skin fluorescence is usually registered in the dark, for instance, in a dark room, a dark chamber or an imaging box with a dark curtain [135]. The second is the source of excitation light (Figure 4A). As a rule, it is a UV-A source, such as a Wood’s lamp or a soft-box UV lamp [24,25]. In some cases, light sources with a broader UV range or short-wavelength visible light are used. 

For a narrow band excitation, one uses bandpass filters for cutting off a certain part of the exciting radiation or monochromatic light sources, such as lasers or light-emitting diodes (LED) with a narrow spectral band of the emission [24]. For instance, a N_2_ laser (337.1 nm), a diode laser (405 nm), or a He-Cd laser (441.6 nm) may be used [10]. The third is the registering device (a photocamera). To cut off the short-wavelength excitation radiation, a light filter is installed in front of the camera lens [23,24,41,107,131].

**Figure 5:**
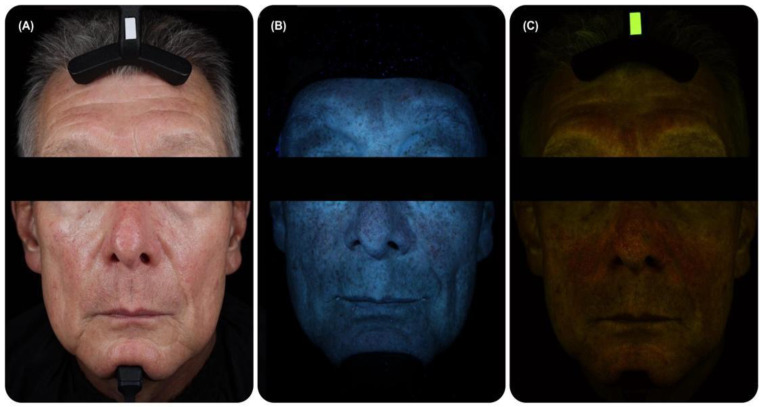
The full-face images obtained by a VISIA-CR 5th gen system (Canfield Scientific, Inc., Parsippany, NJ, USA) in three different modes (unpublished). Bright field white LED lighting; camera: EOS R5 (Canon, Tokyo, Japan) (**A**). Fluorescent mode; camera: EOS R8 with a 50 mm lens without filter (Canon, Tokyo, Japan); excitation: two Multiblitz profilux 600 flash systems with Baader U-Venus filters, 350 ± 60 nm (Multiblitz, Cologne, Germany) (**B**). Fluorescent mode; camera: EOS R series with a with a cut-off short wavelength (orange) filter lens (Canon, Tokyo, Japan); excitation: narrow band blue (405 ± 10 nm) strobes (**C**).

Selection of the correct system of excitation and registration channels is essential. Indeed, different fluorescence patterns can be revealed in different fluorescence registration modes, which are less pronounced in the bright-field mode (Figure 5A). In the blue channel, it is possible to observe regions of hyperpigmentation (Figure 5B). In the mode of long-wavelength detection, red follicular fluorescence is observed (Figure 5C).

Previously, 35 mm film photography had been considered as a “gold standard” in dermatological photography [25]. Then, digital colour cameras (the third component) (Figure 4A) were introduced. The resolution of modern digital cameras far exceeds that of the 35 mm film cameras (4096 × 2736 pixels ≈ 11.2 megapixels) [25,45]. Common models, such as the VS 200E charge-coupled device (CCD) intensified camera (Video Scope International, Sterling, VA, USA) [107], the Coolpix 8400 CCD camera (Nikon, Tokyo, Japan) [120,135], the E2N CCD camera (Nikon, Tokyo, Japan) [41], the CAMEDIA E-20N CCD camera (Olympus Optical Co., Tokyo, Japan) [125], and the Canon CCD cameras (Canon Marketing Japan Inc., Tokyo, Japan) [140], have been mentioned in the FP research. FP has also been registered by digital video cameras (video imaging) [90]. The registration of FP is also possible by modern smartphones. Peris Fajarnés et al. [145] used an iPhone X smartphone (Apple, Cupertino, CA, USA) with a resolution of 12 megapixels. These devices have a number of obvious advantages in comparison with film cameras, e.g., automatic colour calibration and ease of storage and transfer of data [24]. Nevertheless, film cameras, such as the Minolta X-700 camera ((Minolta Corporation, Ramsey, NJ, USA) [131], are also used in recent research. 

### 4.2. Standardised Registration Systems

Over the years, standardised systems for dermatological imaging, including skin FP, have been actively implemented. A number of such systems were patented (e.g., US patents No. 6603552, 6961517, 6922523, 10/008753, 7738032, 8290257) [146,147,148,149,150,151]. Their obvious advantages are fixed lighting regimes and parameters of geometry and optics (distances between the sample, the source of the excited light and the camera, position of the body, magnification, light filters, etc.). Some registered systems specialised in acne detection are equipped with a 405 nm light source for the selective excitation of *C. acnes* porphyrins [24]. This is important for obtaining reproducible results. Furthermore, they usually allow additional images to be taken in the natural bright-field, cross-polarised or parallel-polarised modes for a more detailed analysis of the skin. We categorise these systems into two groups based on the types of skin photographs they register (Table 1).

The first group is developed for the registration of FPs of whole parts of the body, e.g., face (so-called clinical images) (Figure 4B and Figure 5). A vivid example of such equipment, widely used in published articles, is VISIA-CR (Canfield Scientific, Inc., Parsippany, NJ, USA) [89,117,119,121,163]. (Table 1) It was adopted for facial images (Figure 1A and Figure 4B), but it has also been applied to other parts of the body, e.g., hands [150]. It is claimed as a popular tool for dermatological and cosmetic applications as well as for patient education [152,164]. In addition, it is the most popular with researchers compared to analogues (according to the Google Scholar search). The UV-A model of VISIA-CR excites fluorescence in the UV-A range (peak at 365 nm) and detects it in the whole visible range. The narrow band blue model is equipped with light filters for the blue-light excitation of skin fluorescence and its detection in the blue, red, and green channels [164]. It is possible to obtain frontal and side photographs of the face [164]. There are a few other commercially available devices such as OBSERV 520, BS-3800 Skin Analyzer, and DyaDerm Expert, used in scientific research, medical and cosmetological diagnostics, as well as in education, Their excitation and emission characteristics are summarised in Table 1.

The second group refers to the images of the small fragments of the skin captured with or without magnification—so-called dermoscopic images (Figure 1B and Figure 4C). They are highly standardised and characterised by high resolution. Dermoscopic images are taken by special devices called dermoscopes [165]. In a dermoscope, light passes to illuminate the skin, and the image of the skin is viewed or captured through the aperture. The aperture works in conjunction with the dermoscope’s magnifying lenses. The relationship between the aperture size and the focal length of the lenses determines the magnification (usually from ×10 to ×20) and the depth of field. This magnification allows for detailed examination of skin lesions and structures that are not visible to the naked eye. The dermoscope is equipped with a system of LEDs for uniform illumination of the skin fragment. Modern dermoscopes often feature digital cameras for capturing high-resolution images and videos [165]. These digital images can be stored, analysed, and compared over time to monitor changes in skin lesions [166]. Dermoscopes are portable devices that are designed to be comfortable to hold [165]. In addition to the common modes of the natural bright-field and cross-/parallel-polarised light, a few commercially available dermoscopes function in the mode of the registration of skin fluorescence. For this purpose, fluorescence is induced by a UV or 405 nm excitation by LEDs [165]. The Visioscan and Visiopor devices designed by Courage & Khazaka (Table 1) are generally considered the “Gold Standard” for dermoscopic FP and are widely used in basic and applied research [1,42,90,108,127,128,158]. The DL 5 smartphone dermoscope (Table 1), released in 2023, has become popular among researchers and has been mentioned in recent publications, e.g., [40,167,168,169]. The results obtained using the Smart Skin Care dermoscope (Table 1) were successfully published [131]. Nevertheless, devices of other manufacturers are also commercially available and useful in scientific research and clinical practice (Table 1). Some models of dermoscopes are constructed as “digital magnifying glasses” (e.g., Lumio 2 or Optima 3-in-1) equipped with UV LED(s) for the real-time examination of skin fluorescence without image capturing (Table 1).

It is reasonable to mention some modifications of the existing dermoscopic systems for FP registration for a narrow range of specialised tasks. In particular, they may be adopted for acne analysis. FF, related to acne lesions, is emitted in the orange–red range, whereas a significant fraction of skin fluorescence lies in the blue region of the spectrum. This blue background obstructs the observation of luminous follicles. To separate the desired signal, special long-wavelength pass filters are used. Indeed, the use of devices equipped with (>515 nm) [131,145] or (>530 nm) [145] long-wavelength filters enhance acne observation. Obviously, images captured under these conditions cannot be used for the analysis of blue fluorescence and accurate delineation of the pigment spots.

### 4.3. Towards the IoT Technologies for FP Registration

Many modern devices for FP registration come with interfaces for connecting to computers or mobile devices. Some are integrated with software that can analyse the captured images, assist in diagnosing skin conditions, and manage patient records [165,166]. This software often includes features for lesion tracking, image enhancement, and pattern recognition (see below). Recent advances in the development of FP registration lie in the integration of the devices embedded with sensors and processing software with a network, which is commonly termed the Internet of Things (IoT). The IoT allows these devices to collect and share data, enabling smarter, more efficient, and often automated systems.

The Wi-Fi connectivity gives wide opportunities for image transfer and storage, integration with medical software, enhanced workflow, and online diagnostics [166]. It is especially important for small portative dermoscopes. For instance, the Beiersdorf AG company proposes the Skinly IoT solution for capturing dermoscopic images, including fluorescent ones. Skinly includes the device (dermoscope) (Table 1), a smartphone application, and the AI pipeline for the automated determination of skin parameters from the images. Data are transmitted to an internet server using Wi-Fi, and authorised research personnel can access it [158]. Such a system provides opportunities for at-home analysis, which is a worthy alternative to expensive and time-consuming diagnostic tools and visiting clinics [158]. Similarly, the DermLite DL 5 (Table 1) dermoscope can be connected to a smartphone camera, and photographs can be stored and captured via a DermLite application [161]. Data from the Smart Skin Care dermoscope (Table 1) are transferred to a cloud and can be accessed on a smartphone using its application through a Wi-Fi connection [162]. It can expand the frontiers in dermatology and cosmetology by promoting research with higher volumes of datasets, better compliance, and quicker data processing. Moreover, the systems, such as Skinly, DermLite DL or Smart Skin Care 5 enable monitoring anywhere, anytime, and by anyone [158,161,162].

## 5. Data Analysis with Traditional Computer Vision Approaches 

Prior to the widespread adoption of digital cameras, skin fluorescence was qualitatively assessed by direct observation under an excitation source or in film photographs. It was possible to detect obvious local skin defects, such as pigment spots, acne, or psoriatic lesions [21,25,30,38,88,90,117]. McGinley et al. [122] used a grading scale for FF: it was rated as negative, weak, moderate, or intense. The visual observation of FF on photographs correlated with the integral intensity in the red region of the spectrum [118].

Digitalisation has created vast opportunities for the quantitative analysis of FP. While fluorescence signals in spectral data are proportional to the emitted light quanta at specific wavelengths, image analysis relies on subjective colorimetric parameters inferred from human eye sensitivity. To bridge the gap between spectroscopy and colorimetry in skin fluorescence analysis, perceived luminance or brightness (*L*) can be introduced [170] (Figure 4D). Typically, pixel brightness on a digital FP is linearly related to fluorescence intensity (*F*) as follows

(6) L=αF+β

where *α* and *β* are constants [171]. On the other hand, *L* can be represented as

(7) $$L=\left\{R,G,B\right\}\cdot \vec{s}$$

where *R*, *G* and *B* are colour parameters in the RGB Euclidean space and $\vec{s}=\left\{s_{R},s_{G},s_{B}\right\}$ is the vector of three parameters dependent on the perception of the human eye to the red, green, and blue light, respectively [170]. The vector $\left\{R,G,B\right\}$ characterises the colour of each pixel in the digital FF. Thus, the perceived brightness [41,105], as well as *R*, *G*, and *B* values separately [89,145,163], can be used as measures of fluorescence intensity on the images. As a rule, *R*, *G*, and *B* range from 0 to 255; often, normalised values *R*′ = *R*/255, *G*′ = *G*/255, and *B*′ = *B*/255 are used in calculations [170].

The greatest successes were achieved in the analysis of FF. Researchers measure different parameters from FP: the number of fluorescent follicles, their area and diameter, total area in the region of interest (ROI), fraction of the area occupied by the follicles in the ROI, and the integral brightness of the follicles [41,42,117,120,127,135]. The most trivial way is the study of FF in RGB images [135] (Figure 4D). However, because follicular porphyrins emit red light, it is reasonable to decompose the image and extract the R component to increase the performance of the analysis [145]. Advanced algorithms for FP processing include a series of operations that should be applied to an image for its segmentation.

Son et al. [135] proposed the image analysis approach optimised for digital FP. FF was revealed in binary images by an automatic threshold value determined by Otsu’s method [172]. White and red FF were detected separately. In this work, the correlation coefficient between the automatically and manually detected number of fluorescent follicles in images was 0.947 (Table 2).

Khongsuwan et al. [163] reported that the method follows these image processing steps: first, a UV image is cropped to select the ROI. The cropped RGB FP is then resized to a suitable size and converted to a grayscale image, characterised by a single parameter *Grey,* which is a linear combination of R, G, and B. Finally, the extended maxima transform, which is the regional maxima of the H-maxima transform, is applied to count the number of *C. acnes* points. Experimental results demonstrate that the method achieves an accuracy of approximately 83.75%, a sensitivity of 98.22%, and a precision of 85.04%. This approach significantly reduces the time required for analysing acne points (Table 2). Grayscale imaging was also used for AGE detection [105]. The study of FP captured with a blue filter (450–500 nm) showed a correlation between the analytically measured content of advanced glycation end-products and their content as measured by brightness analysis in a certain ROI.

The representation of colours in the RGB space has several limitations. Therefore, other spaces were utilised (Figure 4D). Wu et al. [131] described a segmentation algorithm of FF in the HSV colour space [161]. The HSV components are hue (*H*), saturation (*S*), and value (*V*), which are defined as

(8) $$V=\max\left(R^{\prime };G^{\prime };B^{\prime }\right),$$

(9) $$S=\left\{\begin{array}{c} 0;\;\;\;\;\;\;\max\left(R^{\prime };G^{\prime };B^{\prime }\right)=0\;\\ \frac{\max\left(R^{\prime };G^{\prime };B^{\prime }\right)-\min\left(R^{\prime };G^{\prime };B^{\prime }\right)}{\max\left(R^{\prime };G^{\prime };B^{\prime }\right)};\;\max\left(R^{\prime };G^{\prime };B^{\prime }\right)\neq 0 \end{array}\right.$$

(10) $$H=\left\{\begin{array}{c} \begin{array}{c} 0{}^{\circ};\;\;\;\;\;\;\max\left(R^{\prime };G^{\prime };B^{\prime }\right)=\min\left(R^{\prime };G^{\prime };B^{\prime }\right)\\ 60{}^{\circ}\times \frac{G^{\prime }-B^{\prime }}{\max\left(R^{\prime };G^{\prime };B^{\prime }\right)-\min\left(R^{\prime };G^{\prime };B^{\prime }\right)};\;\max\left(R^{\prime };G^{\prime };B^{\prime }\right)=R\prime \end{array}\\ 60{}^{\circ}\times \left(\frac{B^{\prime }-R^{\prime }}{\max\left(R^{\prime };G^{\prime };B^{\prime }\right)-\min\left(R^{\prime };G^{\prime };B^{\prime }\right)}+2\right);\;\max\left(R^{\prime };G^{\prime };B^{\prime }\right)=G\prime \\ 60{}^{\circ}\times \left(\frac{R^{\prime }-G^{\prime }}{\max\left(R^{\prime };G^{\prime };B^{\prime }\right)-\min\left(R^{\prime };G^{\prime };B^{\prime }\right)}+4\right);\;\max\left(R^{\prime };G^{\prime };B^{\prime }\right)=B^{\prime } \end{array}\right.$$

Using HSV images, the parameters of FF were determined by “mouse click programming”. Only H and V were measured and analysed because S was not associated with FF. This algorithm is applicable for the calculation of total intensity of fluorescence, area of fluorescent regions, and mean intensity of fluorescence in the ROI. The verification results for these parameters were 71%, 72%, and 88%, respectively [131] (Table 2).

## 6. Analysis of Skin Fluorescence Images by AI Algorithms

### 6.1. Possibilities of the Use AI for the Analysis of FP

State-of-the-art FP evaluation lies in the field of automated image analysis by AI. AI is a branch of computer science that focuses on creating smart machines capable of performing tasks that typically require human intelligence [173]. In biomedical studies, AI plays a crucial role by analysing vast datasets of medical information, such as patient records, genomic data, and medical images [174,175,176,177,178]. They give an opportunity for the fast analysis of large amounts of data with satisfied accuracy as well as promote the development of personal medicine using portative devices and mobile applications for skin monitoring [158,174,179,180,181,182,183]. The data on the use of AI for automated FP analysis are poor. However, the approaches developed for the bright-field skin images seem to be applicable in the case of fluorescence. They are listed in the next paragraph.

Alamdari et al. [184] proposed a two-level k-means clustering method for image segmentation to detect acne lesions, achieving an accuracy of about 70%. They also employed machine learning (ML) algorithms for acne classification. The fuzzy c-means method was used to distinguish acne scarring from active inflammatory lesions, with an accuracy of 80%, while the support vector machine achieved an accuracy of 66.6%. Fuzzy c-means clustering was particularly effective in differentiating acne from normal skin with a 100% accuracy rate. Khan et al. [185] introduced an acne segmentation method based on fuzzy c-means clustering, transforming RGB images into different colour spaces and dividing them into homogeneous regions based on colour similarity. Features were extracted from each cluster, and average values were calculated. A new objective function was defined to select the cluster containing lesion pixels based on average feature values. Chang and Liao [186] used support vector machines to classify spots, acne, and normal skin in facial images. Initially, a skin colour detection method was used to identify faces in photographs, which was followed by shape recognition using a contour descriptor. Facial ROIs were extracted, and features such as eyes, eyebrows, mouth, and nostrils were removed in different colour spaces. Segmentation was then performed within each ROI to extract skin lesions. Malik et al. [187] applied high dynamic range theory to enhance acne features in images. For segmentation, they clustered pixels based on the Mahalanobis distance from spectral models of acne vulgaris lesions. Huynh et al. [188] developed the AcneDet system for automatic acne detection and severity grading using facial images taken with smartphones. This system detected four types of acne using a convolutional neural network-based ML model and used a LightGBM ML model for severity grading.

The algorithms mentioned above were developed for the analysis of bright-field images. Peris Fajarnés et al. [145] reported the first (and so far, the only) AI algorithm for FP analysis. They studied FF associated with *C. acnes* in the images obtained using 515 or 530 nm cut-off filters. The R components of the images were extracted for the analysis. The segmentation ML algorithm was based on the k-means clustering. In more detail, the nested k-means was applied: four consecutive procedures of segmentation were performed. The cluster with the highest intensity of the centroid value was assigned to acne. The algorithm was validated by the counting of acne lesions by expert dermatologists. The maximum discrepancy between the number of fluorescent red spots revealed by the algorithms and calculated by the experts was in the range of 4.0–11.2% The effectiveness of the application was more than 90% [145]. Therefore, the algorithm could be considered as a viable tool for automated acne detection. The further implementation of advanced AI algorithms, such as deep neural networks, for FP analysis could be the subject of further research.

The use of AI for the analysis of FPs of the skin offers several promising perspectives. It could enhance the accuracy and consistency of measurements [174,180]. It will improve automation and efficiency in terms of time-saving and large-scale screening. AI-powered systems can be integrated into wearable devices or mobile applications, allowing for the continuous monitoring of skin conditions and early detection of abnormalities [174,180]. AI enables remote diagnostics, allowing patients to receive evaluations and recommendations without the need for frequent in-person visits, which is especially beneficial in remote or underserved areas [158]. It will give an opportunity for integration with other technologies, such as IoT or combine data from multiple imaging modalities (e.g., hyperspectral imaging), enhancing diagnostic capabilities. Furthermore, it is suitable for the development of personalised medicine [174,175,176,180]. AI can analyse patient data to provide personalised treatment recommendations, considering individual variations in skin type, condition severity, and response to treatments [174,177,178].

### 6.2. Possible Legal and Ethical Limitations of the Use of AI in Skin Fluorescent Imaging

Ethical considerations are vital in the integration of AI technologies in dermatology, particularly in the analysis of FP [80,174,176,180]. As AI algorithms become more prevalent in clinical practice, upholding ethical principles such as transparency, fairness, privacy, and accountability is essential [80,174]. Different countries may establish distinct ethical frameworks and regulations for AI shaped by their unique cultural, legal, social, and political environments. These frameworks often reflect the values, priorities, and concerns specific to each nation [80]. Consequently, although there are ongoing global discussions about AI ethics and principles, ethical guidelines and regulatory approaches to AI can vary significantly between countries. For instance, the FDA and the European Medicines Agency (EMA) are actively developing guidelines and protocols for the safe, effective, and qualitative use of devices for registering skin images, including FP, in the USA and EU, respectively [174,175].

Although the ethical issues surrounding FP in cosmetic and skincare research have not been discussed in detail, they can generally be summarised as follows. AI algorithms may exhibit bias if they are trained on non-representative datasets or if incorrect measurements or labelling are involved [80,174]. Georgievskaya et al. [80] noted the potential issue of label bias in FP, as the skin fluorescence signal depends on skin tone; therefore, measured parameters may vary among individuals with different skin colours.

AI systems require access to large datasets, which often include sensitive personal health information [167]. This raises concerns regarding compliance with privacy laws such as the General Data Protection Regulation (GDPR) in the EU and the Health Insurance Portability and Accountability Act (HIPAA) in the USA [174]. Strict data anonymisation, secure storage, and sharing protocols must be followed to protect patient information [174,176,180].

Legal considerations are crucial in the implementation of AI in dermatology. Given the relatively low attention paid by regulatory bodies to fluorescent imaging of the skin, it remains an ongoing challenge in the current legal framework governing dermatology and cosmetology.

## 7. FP as an Alternative or Complement to Bright-Field Imaging

FP offers several advantages over traditional bright-field imaging, particularly in the context of medical diagnostics, research, and the imaging of skin tissues. Specifically, FP provides high contrast and specificity by using skin fluorophores to highlight molecular targets [29,40]. As a result, this method is sensitive to molecular changes, enabling the early detection of skin pathologies that may not be visible in bright-field images [29,40]. It provides potential for diagnosing skin lesions, such as melanoma, psoriasis, pityriasis versicolor, vitiligo, eczema, porokeratosis, Grover's disease, or progressive macular hyperpigmentation, (extensively reviewered in [40,174]). AI algorithms can be applied to identify patterns and abnormalities in FP with greater precision [145].

In contrast, bright-field photography produces natural, full-colour images of the skin, capturing visible features such as colour, texture, and structure. It is widely used and well understood, making it easily accessible and simple to integrate into clinical practice [45]. At the same time, FP typically requires more expensive equipment, and the complexity of fluorescent images can make them harder to interpret without advanced analysis techniques or AI support [40]. Furthermore, FP is not free from limitations, e.g., including the difficulty of distinguishing melanin from haemoglobin, challenges in assessing uneven surfaces, and the presence of artifacts [40,46].

Collectively, bright-field imaging is well established in dermatology with a large body of existing data and trained professionals [174,176,180]. In contrast, FP imaging requires specialised datasets for AI training, which are not as readily available as optical image datasets. Numerous datasets of bright-field dermoscopic images exist, such as the ISIC [189], HAM10000 [190], and MED-NODE [191] dataset, most of which focus on skin pathologies like melanoma. The accuracy, sensitivity, and specificity of classification of skin cancers by the bright-field image analysis are in the ranges of 73.7–97.5%, 73.3–100%, and 74.0–96.7%, respectively [174,176,180]. However, there are currently no publicly available datasets specifically focused on FP. Generating new datasets of fluorescent skin could become a crucial area for the advancement of AI technologies in this field.

## 8. Conclusions

FP is a powerful method for dermatology, cosmetology and skincare. Its theoretical basis lies at the molecular level. The main skin fluorophores include fibrillar proteins (collagen, keratin and elastin), nucleotides, and porphyrins. Skin fluorescence analysis—and FP in particular—has different applications: assessment of sunscreen effectiveness, hyperpigmentation analysis, detection of AGEs and estimation of their levels, evaluation of malignant skin states by special methods, fluorescence decay analysis, revealing acne lesions, and studies of psoriatic plaque. Special clinical imaging and dermoscopic systems are used for the registration of digital FP. Digitalisation gives great opportunities for their analysis. Traditional computer vision methods are used for the analysis of RGB or HSV FP. The processing of FP by AI algorithms is a state-of-the-art method in this field. The implementation of new AI algorithms, particularly neural networks, for FP evaluation could be a potential direction for further research. For the training of machine learning (ML)-based approaches, it is essential to generate new, large datasets of labelled fluorescent images. Moreover, the development of ethical and legal regulations for AI is important for the sustainable development of AI in the analysis of skin FP.

## Figures and Tables

**Table 1:** Commercial devices for registration of fluorescence photographs of the skin. The data on excitation and detection channels are provided. VIS—whole visible range. LED—light emitting diode.

Device	Manufacturer	Excitation	Detection	Reference
Clinical imaging systems				
VISIA-CR (Narrow band model)	Canfield Scientific, Inc., Parsippany, NJ, USA	Xenon flash lightning with blue light filter	Red/Green/Blue	[152]
VISIA-CR (UV-A model)	Canfield Scientific, Inc., Parsippany, NJ, USA	UV-A (peak at 365 nm)	VIS	[152]
OBSERV 520	Sylton Diagnostic Systems, Son, The Netherlands	“Simulated Wood’s”/“True UV”	VIS	[153]
BS-3800 Skin Analyzer	Lumsail Industrial Inc., Shanghai, China	365/405 nm	VIS	[154]
DyaDerm Expert	Biocam GmbH, Regensburg, Germany	409 nm	VIS	[155]
Dermoscopic systems				
Visiopor PP 34 N	Courage & Khazaka, Cologne, Germany	16 LEDs: 375–385	c.a. >500 nm	[156]
Visioscan VC 98	Courage & Khazaka, Cologne, Germany	380–395 nm, peak: 387 nm	VIS	[157]
Skinly	Beiersdorf AG, Hamburg, Germany	4 LEDs: 385 nm	VIS	[158]
Lumio 2	DermLite, Aliso Viejo, CA, USA	365/385/405 nm/ “Wood mode”	“OptiClip”: 405 nm long pass	[159]
Optima 3-in-1	Canfield Scientific GmbH, Bielefeld, Germany	365 nm/“Wood’s light”	VIS	[160]
DermLite DL 5	DermLite Aliso Viejo CA USA	4 LEDs: Peak: 365 nm	VIS	[161]
Smart Skin Care (スマートスキンケア)	IT Access (アイティアクセス), Yokohama, Japan	8 LEDs: 340–405 nm, peak near 377 nm	VIS/315 nm long pass	[131,162]

**Table 2:** Accuracy of algorithms for automated detection of follicular fluorescence on fluorescence photographs. Data sources, type of used images (full face or images obtained by a dermoscope); *n*—size of the dataset.

Reference	Image Source	Approach	Accuracy	*n*
[145]	Facial images	k-means clustering	4.0–11.2% ^a^	54
[163]	Facial images	Extended maxima transform	83.75% ^b^	10
[135]	Facial images	Otsu’s segmentation	0.947 ^c^	29
[131]	Dermoscope	Analysis of colours in HSV space	71% ^b^	10

^a^ Maximum and minimal discrepancy between the number of points counted by the experts and the number of points resulting from the application. ^b^ The overall successful rate of counting the number of fluorescent follicles (average), ^c^ Pearson correlation (*p* < 0.01).

## Data Availability

Not applicable.
